# In this month’s Bulletin

**DOI:** 10.2471/BLT.26.001126

**Published:** 2026-11-01

In the editorial section, Ahmad Reza Hosseinpoor et al. (718) summarize WHO’s work on addressing the inequalities that affect noncommunicable disease prevention and control. Marcelo D’Agostino et al. (719) discuss the implications of governments wishing to pursue digital sovereignty in public health. 

## Benin, Burkina Faso, Niger and Togo

### HIV/AIDS, tuberculosis and malaria 

Casimir Manzengo et al. (760–765) describe WHO regional office coordination of programme support.

## China

### A rural toilet revolution

Zhenyu Zhang et al. (740–749) estimate health and economic impacts.

## India

### Correct use of child growth charts

Seetharaman Narayanan et al. (732–739) point to a design and communication error.

## Global 

### Cross-border transport of infectious substances

Markus Huber et al. (722–731) review the international legal framework.

### Interim medical countermeasures for public health emergencies

Tim Nguyen et al. (750–759) introduce a WHO network.

### Pandemic prevention, preparedness and response

Ong-orn Prasarnphanich et al. (766–771) call for a One Health workforce.

### How to improve use of verbal autopsies

Chalapati Rao et al. (772–774) list developments in physician assignment of cause-of-death.

### Pregnancy and postnatal care recommendations

Mercedes Bonet et al. (775–776) clarify the scope of WHO guidance.

